# Gadd45 stress sensors in suppression of leukemia

**DOI:** 10.18632/oncotarget.26154

**Published:** 2018-09-28

**Authors:** David L. Wiest

**Affiliations:** Blood Cell Development and Function Program, Fox Chase Cancer Center, Philadelphia, PA, USA

**Keywords:** Gadd45, stress, tumor suppressor, CML, AML

The Gadd45 family of proteins (Gadd45a, Gadd45b, and Gadd45g) acts as stress sensors in response to various physiological and environmental stressors, including oncogenic stress [1] Upon sensing stress, Gadd45 can act to regulate numerous cell behaviors such as cell cycle, DNA replication/repair, and survival, and it does so via interaction with a collection of partner proteins, including PCNA, cdk1/cyclinB1, p21, MEKK4, MKK7, and p38 [1]. Notably, Gadd45a is capable of functioning as a tumor promoter or suppressor, depending on context. Indeed, Gadd45a behaves as a tumor suppressor in H-RAS driven models of breast cancer and as an oncogene in Myc-driven tumors [2]. Although members of the Gadd45 family are infrequently mutated in cancer, it should be noted that their reduced expression due to promoter methylation has been observed in several types of human cancers [3]. For example, Gadd45a expression is repressed by activated FLT3 [4] and *Gadd45a* promoter methylation has been shown to be predictive of poor outcomes in AML [5]. Together, these findings suggest that the expression level of Gadd45 proteins may have prognostic value in solid and well as hematologic malignancies.

Chronic Myelogenous Leukemia (CML) has been causally-linked to the Philadelphia chromosome (Ph), which represents a balanced translocation involving chromosomes 9 and 22 that forms the BCR-ABL fusion oncoprotein, an active tyrosine kinase. CML is characterized by progression from an indolent ‘chronic phase’ (CML-CP), characterized by hyperproliferation of mature granulocytes, to the aggressive and fatal ‘blast crisis’ (CML-BC) phase, marked by the clonal expansion of differentiation-arrested immature blasts [reviewed in ref. 6]. Imatinib is a small molecule ABL kinase inhibitor that is highly effective in treating CML-CP patients [reviewed in ref. 6]; however, a substantial number of patients relapse due to development of resistance to imatinib therapy, which leads to CML-BC and death within weeks to months. Thus, identification of additional genetic aberrations that play a role in CML progression is of the utmost importance, as they may serve not only as prognostic markers, but also as novel therapeutic entry points.

In this issue of *Oncotarget*, Sha *et al*. [7] report that loss of Gadd45b accelerates the development of BCR-ABL driven CML in mice and leads to decreased median survival. The Gadd45b-deficient CML progenitors exhibited increased proliferation and decreased apoptosis, and this was associated with hyper-activation of c-Jun NH_2_-terminal kinase and Stat5. The same group previously reported similar findings for loss of another family member, Gadd45a. Loss of Gadd45a also accelerated the development of BCR-ABL driven CML, and this was associated with enhanced PI3K-AKT-mTOR-4E-BP1 signaling, upregulation of p30C/EBPα expression, and hyper-activation of p38 and Stat5. Importantly, these findings are highly clinically relevant, since Gadd45a expression in human CML patients is high in the indolent, chronic phase of CML, but is markedly downregulated in the aggressive, accelerated phase of CML and blast crisis CML [8]. Collectively, these results provide novel evidence that Gadd45a functions as a suppressor of BCR/ABL driven leukemia and may serve as a unique prognostic marker of CML progression. Also, these findings provide novel evidence that Gadd45b, like Gadd45a, functions as a suppressor of BCR-ABL driven leukemia, albeit via a different mechanism.

The role of Gadd45 family proteins as tumor suppressors may also be important in other hematologic malignancies as other studies have shown that the repression of Gadd45a by activated FLT3 and GM-CSF receptor mutants contributes to growth, survival and blocked differentiation in both the murine factor-dependent bi-potential myeloid cell line, FDB1, and in two human FLT3-ITD AML cell lines (MV4;11 and MOLM-13) [4]. *Gadd45a* methylation is also a predictor of poor overall survival in a broad spectrum of acute myeloid leukemias, and is associated with *IDH1/2* and *DNMT3A* mutations [5].

Nevertheless, many questions remain to be addressed before the full potential of these findings can be realized. 1) How do Gadd45 proteins sense BCR-ABL leukemic signaling? 2) Do they function upstream or downstream of the BCR-ABL fusion? 3) Can Gadd45 upregulation mitigate Imatinib resistance or attenuate CML progression upon development of resistance? 4) Can pharmacologic inhibition of Gadd45 regulated signaling pathways be exploited in Imatinib resistant CML and in FLT3 and MLL9-AF9 AML? Answers to these questions may significantly inform the management of CML by predicting and attenuating resistance to Imatinib.
